# Influence of the ordering of impurities on the appearance of an energy gap and on the electrical conductance of graphene

**DOI:** 10.1038/s41598-018-26925-0

**Published:** 2018-06-14

**Authors:** S. P. Repetsky, I. G. Vyshyvana, S. P. Kruchinin, Stefano Bellucci

**Affiliations:** 1 Institute of High Technologies, Taras Shevchenko National University of Kiev, 4-g, Academician Glushkov Ave., Kiev, 03022 Ukraine; 2 Bogolyubov Institute for Theoretical Physics, NASU, Kiev, 03143 Ukraine; 3 INFN-Laboratori Nazionali di Frascati, Via E. Fermi, 40, 00044 Frascati, Italy

## Abstract

In the one-band model of strong coupling, the influence of substitutional impurity atoms on the energy spectrum and electrical conductance of graphene is studied. It is established that the ordering of substitutional impurity atoms on nodes of the crystal lattice causes the appearance of a gap in the energy spectrum of graphene with width *η*|*δ*| centered at the point *yδ*, where *η* is the parameter of ordering, *δ* is the difference of the scattering potentials of impurity atoms and carbon atoms, and *y* is the impurity concentration. The maximum value of the parameter of ordering is ${{\boldsymbol{\eta }}}_{{\boldsymbol{\max }}}{\boldsymbol{=}}{\bf{2}}{\boldsymbol{y}}{\boldsymbol{,}}\,{\boldsymbol{y}}{\boldsymbol{\le }}{\bf{1}}/{\bf{2}}$. For the complete ordering of impurity atoms, the energy gap width equals ${\bf{2}}{\boldsymbol{y}}{\boldsymbol{|}}{\boldsymbol{\delta }}{\boldsymbol{|}}$. If the Fermi level falls in the region of the mentioned gap, then the electrical conductance ${{\boldsymbol{\sigma }}}_{{\boldsymbol{\alpha }}{\boldsymbol{\alpha }}}{\boldsymbol{\to }}{\bf{0}}$ at the ordering of graphene, i.e., the metal–dielectric transition arises. If the Fermi level is located outside the gap, then the electrical conductance increases with the parameter of order *η* by the relation ${{\boldsymbol{\sigma }}}_{{\boldsymbol{\alpha }}{\boldsymbol{\alpha }}}{\boldsymbol{ \sim }}{{\boldsymbol{(}}{{\boldsymbol{y}}}^{{\bf{2}}}{\boldsymbol{-}}\frac{{\bf{1}}}{{\bf{4}}}{{\boldsymbol{\eta }}}^{{\bf{2}}}{\boldsymbol{)}}}^{{\boldsymbol{-}}{\bf{1}}}$. At the concentration ${\boldsymbol{y}}{\boldsymbol{=}}{\bf{1}}{\boldsymbol{/}}{\bf{2}}$, as the ordering of impurity atoms *η* →1, the electrical conductance of graphene ${{\boldsymbol{\sigma }}}_{{\boldsymbol{\alpha }}{\boldsymbol{\alpha }}}{\boldsymbol{\to }}{\boldsymbol{\infty }}$, i.e., the transition of graphene in the state of ideal electrical conductance arises.

## Introduction

In recent times, a special attention has been paid to the possibility of a targeted modification of graphene with the help of purposely introduced impurities, formed defects, and atoms or chemical functional groups deposited on a surface. In this case, wide possibilities to change the physical properties of graphene are opened, due to the controlled introduction of impurities by the method of ion implantation. In such a way, graphene becomes a basic system that generates a new class of functional materials. Sometimes, such materials find unexpected applications: from nanoelectromechanical systems to hydrogen-accumulating systems. Of course, the main hopes are set upon graphene, because it has all possibilities to become, in the nearest future, a substitute of silicon in electronic devices, which will allow one to significantly enhance the level of their miniaturization and to increase the working frequencies. The quasirelativistic spectrum of charge carriers determines the unique properties of graphene and, simultaneously, hampers the use of graphene in field-effect transistors due to the absence of a gap in its spectrum. It is known that the impurities can lead to the appearance of such a gap, whose width depends on the type of impurities and their concentration.

The recent investigations of the energy spectrum of graphene are based on the density functional theory. It is worth to note the advantages of this theory related to the self-consistent meta-generalized gradient approximation within the projector-augmented-wave method^1^ which is realized with softwares WASP and GAUSSIAN^1^. The numerical calculations made within the method have demonstrated the appearance of a gap in the energy spectrum of graphene caused by the presence of an impurity. However, in order to clarify the nature of this effect, it is necessary to supplement the mentioned numerical calculations by analytic studies of the influence of impurities on the energy spectrum and properties of graphene.

Recently, new 2D-material nanomeshes based on Moiré graphene/hexagonal boron nitride bilayers were predicted, on the basis of first-principle calculations within the framework of density functional theory, with standard norm-conserving pseudo-potentials, flexible numerical LCAO double zeta + polarization orbital basis sets^2^. The energy band gap in the bilayer graphene/hexagonal boron nitride material then increases, due to symmetry breaking in the curvature graphene component and due to the charge transport at the toroidal border of the hole compared with a similar graphene mesh^2^. Also, in earlier work, the complex structure based on the graphene monolayer and the twisted BN monolayer was investigated^3^. The changes in the electronic structure during the hydrogen adsorption at low concentration were considered. It was found that the presence of the BN layer did not impact significantly on the electronic structure and that only the H-atom adsorption on the boron atoms could open the band gap in the layered structuré. An investigation of the dependence of the band gap on the hydrogen concentration on the Moire surface was made. Upon increasing the hydrogen concentration, the value of the band gap was increased up to 0.5 eV^3^.

The effects generated by the Moiré superlattice of a van-der-Waals heterostructure of graphene grown on ultrathin layers of hexagonal boron nitride (h-BN) were studied in review^4^. There, the effects of the opening of a gap in the energy spectrum, which are connected with the breaking of a symmetry of sublattices due to the appearance of internal mechanical stresses, were analyzed. The influence of a magnetic field on the energy spectrum of a heterostructure was studied as well^4^.

In work^5^ in the frame of the density functional theory with the use of the method of pseudopotential, the electron structures of an isolated monolayer of graphene, two and three layers of graphene, and graphene grown on ultrathin layers of hexagonal boron nitride (h-BN) were calculated. It was shown that, for graphene grown on an h-BN monolayer, the energy gap 57 meV in width arises. By an analogous method, graphene with aluminum, silicon, phosphorus, and sulfur impurities was studied in work^6^. It was shown^6^ that graphene with a 3-% phosphorus impurity has a gap 0.67 eV in width.

By using the software QUANTUM-ESPRESSO, the authors of work^7^ demonstrated the possibility to open a gap in the energy spectrum of graphene at the introduction of impurity atoms of boron and nitrogen (gap 0.49 eV in width), as well as impurity atoms of boron and atoms of lithium adsorbed on the surface (gap 0.166 eV in width).

Works^8–10^ present the theory of reconstruction of the spectrum of graphene, which occurs at a growth in the concentration of point-like impurities, and foresee the possibility of the metal–dielectric transition in such a system. The course of the reconstruction of the spectrum, which was predicted on the basis of the analytic calculations, was confirmed with the help of a numerical experiment. The latter showed the existence of a quasigap filled with localized states and demonstrated the dominating role of the scattering by pairs and triples of impurity centers in the localization.

In works^11–16^, the influence of impurity atoms or atoms adsorbed on the surface on the electron structure and electrical conductance of graphene was studied. The numerical calculations were carried out in the frame of the quantum-mechanical formalism. In work^17^, the influence of the ordering of atoms on the energy spectrum and the electrical conductance of an alloy was studied analytically in the above-mentioned one-band model. It was found^17^ that, at the long-range ordering of the alloy, a gap appears in the energy spectrum of electrons. Its width is equal to the difference of the scattering potentials of the components of the alloy. The appearance of the metal–dielectric transition was established in the case where the Fermi level enters into the indicated gap at the long-range ordering of atoms in the alloy. However, no explanation about the nature of the influence of the ordering of impurities on the appearance of a gap in the energy spectrum of graphene is available until now.

It is worth to note that, at the appearance of a gap in the energy spectrum of graphene in the case where the Fermi level enters into the indicated gap, the velocity of an electron on the Fermi level can decrease. This causes a decrease in the mobility and electrical conductance of electrons, which can deteriorate the functional characteristics of graphene, which would be used as a material for field-effect transistors instead of the traditional materials based on silicon and germanium. The influence of disordered impurities on the electrical conductance of graphene was investigated in works^18,19^. In particular, it was established in work^18^ that the presence of impurities can cause a significant decrease in the electrical conductance of graphene.

The authors of the above-indicated works studied the effects related to the appearance of a gap in the energy spectrum of a heterostructure of graphene grown on ultrathin layers of hexagonal boron nitride (h-BN)^2–5^ and graphene with an impurity adsorbed on the surface and with a substitutional impurity^6–10^. In the present work, we will demonstrate one more cause for the appearance of a gap in the energy spectrum of graphene that is related to the ordering of a substitutional impurity. We will study the influence of the ordering of a substitutional impurity on the conductivity of graphene.

In order to clarify the influence of the ordering of substitutional impurities on the energy spectrum and electrical conductance of graphene, we use the Lifshitz simple one-band model.

The Hamiltonian describing the one-electron states of graphene with substitutional impurity atoms can be presented in the form^17^

1 $$H=\sum _{in}|in\rangle {v}_{in}\langle in|+\sum _{\begin{array}{c}in,i^{\prime} n^{\prime} \ne in\\ n,n^{\prime} \ne n\end{array}}|in\rangle {h}_{in,i^{\prime} n^{\prime} }\langle i^{\prime} n^{\prime} |,$$

where ${h}_{in,i^{\prime} n^{\prime} }$ is a matrix element (hopping integral) of the Hamiltonian nondiagonal in the Wannier representation, which is independent of the random distribution of atoms in the accepted approximation of diagonal disorder; ${v}_{in}$ is the diagonal matrix element taking value ${v}^{A}$ or ${v}^{B}$ depending on that which atom (*A* or *B*) is located at the node *in*; *i* is the number of a sublattice, and *n* is the number of a node of the sublattice.

In formula (1), we now add and subtract the translationally invariant operator $\sum _{in}|in\rangle {\sigma }_{i}\langle in|$, where ${\sigma }_{i}$ is the diagonal matrix element of the Hamiltonian of some effective ordered medium (coherent potential), which depends on the number of a sublattice. As a result, the Hamiltonian of graphene can be presented in the form

2 $$\begin{array}{rcl}H & = & \tilde{H}+\tilde{V}\\ \tilde{H} & = & \sum _{in}|in\rangle {\sigma }_{i}\langle in|+\sum _{\begin{array}{c}in,i^{\prime} n^{\prime} \ne in\\ n,n^{\prime} \ne n\end{array}}|in\rangle {h}_{in,i^{\prime} n^{\prime} }\langle i^{\prime} n^{\prime} |\\ \tilde{V} & = & {\sum }_{in}{\tilde{v}}_{in},\,\,\,{\tilde{v}}_{in}=|in\rangle ({v}_{in}-{\sigma }_{i})\langle in|.\end{array},$$

In Eq. (2), ${\sigma }_{i}$ are potentials of the effective medium (coherent potentials). The values of coherent potentials will be specified in what follows.

The retarded Green function of graphene, which is an analytic function of the complex energy *z* in the upper half-plane, reads

3 $$G(z)={(z-H)}^{-1}.$$

The Green function satisfies the Dyson equation

4 $$G=\tilde{G}+\tilde{G}\tilde{V}G,$$

where

5 $$\tilde{G}={(z-\tilde{H})}^{-1}$$

is the Green function for the effective Hamiltonian $\tilde{H}$ in formula (2). The *T*-matrix of the scattering by a random potential is determined by the relation^17^

6 $$G=\tilde{G}+\tilde{G}T\tilde{G}$$

and satisfies the equation

7 $$T=\tilde{V}+\tilde{V}\tilde{G}T,$$

which follows from formulas (4) and (6).

The scattering matrix *T* takes the form

8 $$T={\sum }_{in}{T}_{in}$$

Substituting relation (2) for the scattering potential $\tilde{V}$ and formulas (8) in (7), we get the *T*-matrix as an infinite series^17^:

9 $$T=\sum _{({n}_{1}{i}_{1})}{t}^{{n}_{1}{i}_{1}}+\sum _{({n}_{1}{i}_{1})\ne ({n}_{2}{i}_{2})}{T}^{(2){n}_{1}{i}_{1},{n}_{2}{i}_{2}}+\mathrm{....}$$

Here,

10 $${T}^{(2){n}_{1}{i}_{1},{n}_{2}{i}_{2}}={[I-{t}^{{n}_{1}{i}_{1}}\tilde{G}{t}^{{n}_{2}{i}_{2}}\tilde{G}]}^{-1}{t}^{{n}_{1}{i}_{1}}\tilde{G}{t}^{{n}_{2}{i}_{2}}[I+\tilde{G}{t}^{{n}_{1}{i}_{1}}],$$

where

11 $${t}^{ni}={[I-{\tilde{v}}_{in}\tilde{G}]}^{-1}{\tilde{v}}_{in-}$$

is scattering operator on the same site, and *I* is the identity operator. The terms of series (9) describe the processes of multiple scattering of electrons by one center and by the clusters of two, three, etc. scattering centers.

Neglecting the contribution of the processes of scattering by the clusters of three and more atoms, which are small in some parameter^17^, we present the density of one-electron states of graphene in the form

12 $$\begin{array}{rcl}g({\rm{\varepsilon }}) & = & \frac{1}{v}\sum _{i,{\rm{\lambda }}}{P}^{{\rm{\lambda }}0i}{g}^{{\rm{\lambda }}0i}({\rm{\varepsilon }})\\ {g}^{\lambda 0i}({\rm{\varepsilon }}) & = & -\frac{2}{\pi }\,{\rm{Im}}\,\{\tilde{G}+\tilde{G}{t}^{{\rm{\lambda }}0i}\,\tilde{G}+\sum _{\begin{array}{c}(lj)\ne (0i)\\ {\rm{\lambda }}^{\prime} \end{array}}{P}^{{\rm{\lambda }}^{\prime} lj/{\rm{\lambda }}0i}\\  &  & \times \tilde{G}\,[{t}^{\lambda ^{\prime} lj}+{T}^{(2){\rm{\lambda }}0i,{\rm{\lambda }}^{\prime} lj}+{T}^{(2){\rm{\lambda }}^{\prime} lj,{\rm{\lambda }}0i}]{\tilde{G}\}}_{0i,0i},\end{array}$$

where ${g}^{{\rm{\lambda }}0i}({\rm{\varepsilon }})$ is conditional partial density of states, $\nu =2$ is the number of sublattices of graphene.

Using the Kubo—Greenwood formula^20^ and neglecting the contribution of the processes of scattering by clusters composed of at least three atoms, we get the static electrical conductance of graphene in the form (*T* = 0)^17^:

13 $$\begin{array}{rcl}{\sigma }_{\alpha ,\beta } & = & -\frac{{e}^{2}\hslash }{2\pi {{\rm{\Omega }}}_{1}}\,\sum _{s,s^{\prime} =r,a}\sum _{i}\{{v}_{\beta }{\tilde{K}}_{ss^{\prime} }({v}_{\alpha },\varepsilon )\\  &  & +\,\sum _{\lambda }\{{\tilde{K}}_{s^{\prime} s}({v}_{\beta },\varepsilon ){t}_{s}^{\lambda 0i}(\varepsilon )\,{\tilde{K}}_{ss^{\prime} }({v}_{\alpha },\varepsilon )\,{t}_{s^{\prime} }^{\lambda 0i}(\varepsilon )\\  &  & +\,\sum _{\begin{array}{c}lj\ne 0i,\\ \lambda ^{\prime} \end{array}}{P}^{\lambda ^{\prime} lj/\lambda 0i}[{\tilde{K}}_{s^{\prime} s}({v}_{\beta },\varepsilon )\,{v}_{\alpha }{\tilde{G}}_{s^{\prime} }(\varepsilon ){T}_{s^{\prime} }^{(2)\lambda 0i,\lambda ^{\prime} lj}(\varepsilon )\\  &  & +\,{\tilde{K}}_{s^{\prime} s}({v}_{\beta },\varepsilon ){v}_{\alpha }{\tilde{G}}_{s^{\prime} }(\varepsilon ){T}_{s^{\prime} }^{(2)\lambda ^{\prime} lj,\lambda 0i}(\varepsilon )+{\tilde{K}}_{ss^{\prime} }({v}_{\alpha },\varepsilon ){v}_{\beta }{\tilde{G}}_{s}(\varepsilon ){T}_{s}^{(2)\lambda 0i,\lambda ^{\prime} lj}(\varepsilon )\\  &  & +\,{\tilde{K}}_{ss^{\prime} }({v}_{\alpha },\varepsilon ){v}_{\beta }{\tilde{G}}_{s}(\varepsilon ){T}_{s}^{(2)\lambda ^{\prime} lj,\lambda 0i}(\varepsilon )\\  &  & +\,{\tilde{K}}_{s^{\prime} s}({v}_{\beta },\varepsilon )\,[{t}_{s}^{\lambda ^{\prime} lj}(\varepsilon ){\tilde{K}}_{ss^{\prime} }({v}_{\alpha },\varepsilon ){t}_{s^{\prime} }^{\lambda 0i}(\varepsilon )\\  &  & +\,{t}_{s}^{\lambda ^{\prime} lj}(\varepsilon ){\tilde{K}}_{ss^{\prime} }({v}_{\alpha },\varepsilon ){T}_{s^{\prime} }^{(2)\lambda 0i,\lambda ^{\prime} lj}(\varepsilon )+{T}_{s}^{(2)\lambda ^{\prime} lj,\lambda 0i}(\varepsilon ){\tilde{K}}_{ss^{\prime} }({v}_{\alpha },\varepsilon ){t}_{s^{\prime} }^{\lambda 0i}(\varepsilon )\\  &  & +\,{T}_{s}^{(2)\lambda ^{\prime} lj,\lambda 0i}(\varepsilon ){\tilde{K}}_{ss^{\prime} }({v}_{\alpha },\varepsilon ){T}_{s^{\prime} }^{(2)\lambda 0i,\lambda ^{\prime} lj}(\varepsilon )\\  &  & {{+{T}_{s}^{(2)\lambda ^{\prime} lj,\lambda 0i}(\varepsilon ){\tilde{K}}_{ss^{\prime} }({v}_{\alpha },\varepsilon ){T}_{s^{\prime} }^{(2)\lambda ^{\prime} lj,\lambda 0i}(\varepsilon )]]\}\}}_{0i,0i}|}_{\varepsilon =\mu }(2{\delta }_{ss^{\prime} }-1),\end{array}$$

where ${\tilde{K}}_{ss^{\prime} }({v}_{\alpha },\varepsilon )={\tilde{G}}_{s}(\varepsilon ){v}_{\alpha }{\tilde{G}}_{s\text{'}}(\varepsilon )$, ${\tilde{G}}_{r}(\varepsilon )$ and ${\tilde{G}}_{a}(\varepsilon )$ are the retarded and advanced Green functions, ${t}_{s}^{{\lambda }^{\text{'}}lj}(\varepsilon )$ and ${T}_{s}^{(2)\lambda ^{\prime} lj,\lambda 0i}(\varepsilon )$ are scattering operators determined by the Green function ${\tilde{G}}_{s}(\varepsilon ),$ ${\delta }_{ss^{\prime} }$ is Kronecker’s symbol, $\mu$ is Fermi level, ${{\rm{\Omega }}}_{1}=\nu {{\rm{\Omega }}}_{0}$ is the volume of an elementary cell of graphene, and ${{\rm{\Omega }}}_{0}$ is the volume per atom.

In formulas (12) and (13), ${P}^{\lambda 0i}$ is the probability of the occupation of node 0*i* of the crystal lattice $(i=1,\,2)$ by atoms of the sort$\,\lambda =A,B:$

14 $$\begin{array}{c}{P}^{B01}={y}_{1}=y+\frac{1}{2}\eta \,{P}^{B02}={y}_{2}=y-\frac{1}{2}\eta \,{P}^{A0i}=1-{P}^{B0i}\end{array}$$

where *y* is the concentration of impurity atoms, and *η* is the parameter of atomic ordering.

In formulas (12) and (13), ${P}^{\lambda ^{\prime} lj/\lambda 0i}$ is the probability of the occupation of node *lj* by an atom of the sort $\lambda ^{\prime}$ under the condition that an atom of the sort $\lambda$ occupies node *0i* (the parameter of binary interatomic correlations in the occupation of nodes of the crystal lattice by impurity atoms).

The coherent potential is determined from the condition $\langle {t}^{{n}_{1}{i}_{1}}\rangle =0$. The brackets mean the averaging over the distribution of impurity atoms on nodes of the crystal lattice. The above-mentioned condition yields the equation for the coherent potential^17^:

15 $${\sigma }_{i}=\langle {v}_{i}\rangle -({v}_{A}-{\sigma }_{i}){\tilde{G}}_{0i,0i}(\varepsilon )({v}_{B}-{\sigma }_{i});\,\langle {v}_{i}\rangle =(1-{y}_{i}){v}_{A}+{y}_{i}{v}_{B}.$$

Setting ${v}_{A}=0$ in relations (15), we get

16 $$\langle {v}_{i}\rangle ={y}_{i}\delta ,$$

where

17 $$\delta ={v}_{B}-{v}_{A}$$

is the difference of the scattering potentials of components of graphene.

In the analytic description of the energy spectrum and electrical conductance of graphene, we consider only the first terms in relations (12) and (13), which give the main contribution to the density of states and to the electrical conductance.

The mentioned components can be presented in the form:

18 $$g(\varepsilon )=-\,\frac{2}{\pi \nu }Im\sum _{i}{\tilde{G}}_{0i,0i}(\varepsilon )=-\,\frac{2}{\pi \nu N}Im\sum _{i,{\boldsymbol{k}}}{\tilde{G}}_{ii}({\boldsymbol{k}},\varepsilon ),$$

19 $$\begin{array}{rcl}{\sigma }_{\alpha \alpha } & = & -\frac{{e}^{2}\hslash }{2\pi {{\rm{\Omega }}}_{1}}{\sum _{i}{[{v}_{\alpha }(\tilde{G}(\varepsilon )-{\tilde{G}}^{\ast }(\varepsilon )){v}_{\alpha }(\tilde{G}(\varepsilon )-{\tilde{G}}^{\ast }(\varepsilon ))]}_{0i,0i}|}_{\varepsilon =\mu }\\  & =\, & -{\frac{{e}^{2}\hslash }{2\pi {{\rm{\Omega }}}_{1}N}\sum _{i,{\boldsymbol{k}}}{[{v}_{\alpha }({\boldsymbol{k}})(\tilde{G}({\boldsymbol{k}},\varepsilon )-{\tilde{G}}^{\ast }({\boldsymbol{k}},\varepsilon )){v}_{\alpha }({\boldsymbol{k}})(\tilde{G}({\boldsymbol{k}},\varepsilon )-{\tilde{G}}^{\ast }({\boldsymbol{k}},\varepsilon ))]}_{0i,0i}|}_{\varepsilon =\mu }.\end{array}$$

The operator of the $\alpha$-projection of the velocity of an electron in formula (19) is:

20 $${v}_{\alpha ii^{\prime} }({\boldsymbol{k}})=\frac{1}{\hslash }\frac{\partial {h}_{ii^{\prime} }({\boldsymbol{k}})}{\partial {k}_{\alpha }},$$

where $\,{h}_{ii^{\prime} }({\boldsymbol{k}})$ is the Fourier transform of the hopping integral.

The wave vector in formulas (18) and (19) varies in the limits of the Brillouin zone of graphene.

We calculated $\,{h}_{ii^{\prime} }({\boldsymbol{k}})$ in the approximation of nearest neighbors. The Fourier transform of the Green function in this approximation takes the form

21 $$\begin{array}{c}\begin{array}{cc}{\tilde{{\boldsymbol{G}}}}_{{\bf{11}}}({\boldsymbol{k}},{\boldsymbol{\varepsilon }})=\frac{{\boldsymbol{\varepsilon }}-{{\boldsymbol{\sigma }}}_{{\bf{2}}}}{{\boldsymbol{D}}({\boldsymbol{k}},{\boldsymbol{\varepsilon }})}, & {\tilde{{\boldsymbol{G}}}}_{{\bf{12}}}({\boldsymbol{k}},{\boldsymbol{\varepsilon }})=\frac{{{\boldsymbol{h}}}_{{\bf{21}}}({\boldsymbol{k}})}{{\boldsymbol{D}}({\boldsymbol{k}},{\boldsymbol{\varepsilon }})},\\ {\tilde{{\boldsymbol{G}}}}_{{\bf{21}}}({\boldsymbol{k}},{\boldsymbol{\varepsilon }})=\frac{{{\boldsymbol{h}}}_{{\bf{12}}}({\boldsymbol{k}})}{{\boldsymbol{D}}({\boldsymbol{k}},{\boldsymbol{\varepsilon }})}, & {\tilde{{\boldsymbol{G}}}}_{{\bf{22}}}({\boldsymbol{k}},{\boldsymbol{\varepsilon }})=\frac{{\boldsymbol{\varepsilon }}-{{\boldsymbol{\sigma }}}_{{\bf{1}}}}{{\boldsymbol{\varepsilon }}-{{\boldsymbol{\sigma }}}_{{\bf{2}}}}{\tilde{{\boldsymbol{G}}}}_{{\bf{11}}}({\boldsymbol{k}},{\boldsymbol{\varepsilon }}),\end{array}\\ D({\boldsymbol{k}},\varepsilon )=(\varepsilon -{\sigma }_{1})(\varepsilon -{\sigma }_{2})-{h}_{12}({\boldsymbol{k}}){h}_{21}({\boldsymbol{k}}).\end{array}$$

In the used model, the main contribution to the energy spectrum of electrons in the middle of the zone is given by the values of the wave vector ${\boldsymbol{k}}$, which belong to the domains near Dirac points. The Brillouin zone contains two such domains, where

22 $${h}_{12}({\boldsymbol{k}})={h}_{21}({\boldsymbol{k}})=\hslash {v}_{F}k,$$

${v}_{F}=\frac{3|{\gamma }_{1}|{a}_{0}}{2\hslash }$ is the velocity of the electron on the Fermi level, and ${\gamma }_{1}=(pp\pi )\,{\rm{and}}\,{a}_{0}$ are the hopping integral^21^ and the distance between the nearest neighbors, respectively.

Substituting formulas (21) and (22) in (18) and passing from the summation over the wave vectors ${\boldsymbol{k}}$ to the integration, we get

23 $$\begin{array}{c}{\tilde{G}}_{01,01}\,(\varepsilon )=-\,\frac{{{\rm{S}}}_{1}(\varepsilon -{\sigma }_{2})}{\pi d{\hslash }^{2}{v}_{F}^{2}}ln\sqrt{1-\frac{{w}^{2}}{(\varepsilon -{\sigma }_{1})(\varepsilon -{\sigma }_{2})}},\\ {\tilde{G}}_{02,02}\,(\varepsilon )=-\,\frac{{{\rm{S}}}_{1}(\varepsilon -{\sigma }_{1})}{\pi d{\hslash }^{2}{v}_{F}^{2}}ln\sqrt{1-\frac{{w}^{2}}{(\varepsilon -{\sigma }_{1})(\varepsilon -{\sigma }_{2})}},\end{array}$$

where $w=3|{\gamma }_{1}|$ is the half-width of the energy band of pure graphene, and ${{\rm{S}}}_{1}=3\sqrt{3}{a}_{0}^{2}/2$ is the area of an elementary cell of graphene.

Let us consider the influence of the ordering of atoms on the energy spectrum of electrons of graphene with a substitutional impurity in the limiting case of weak scattering where $|\delta /w|\ll 1$.

In this case, the solution of the system of equations (15), (23) is as follows:

24 $$\begin{array}{c}{\tilde{G}}_{01,01}(\varepsilon )=-\,\frac{{{\rm{S}}}_{1}(\varepsilon -{\sigma ^{\prime} }_{2})}{\pi d{\hslash }^{2}{v}_{F}^{2}}ln\sqrt{1-\frac{{w}^{2}}{(\varepsilon -{\sigma ^{\prime} }_{1})(\varepsilon -{\sigma ^{\prime} }_{2})}},\\ {\tilde{G}}_{02,02}(\varepsilon )=-\,\frac{{{\rm{S}}}_{1}(\varepsilon -{\sigma ^{\prime} }_{1})}{\pi d{\hslash }^{2}{v}_{F}^{2}}ln\sqrt{1-\frac{{w}^{2}}{(\varepsilon -{\sigma ^{\prime} }_{1})(\varepsilon -{\sigma ^{\prime} }_{2})}},\end{array}$$

25 $$\begin{array}{c}{\sigma ^{\prime} }_{1}={y}_{1}\delta -{y}_{1}(1-{y}_{1}){\delta }^{2}\frac{{{\rm{\Omega }}}_{1}(\varepsilon -{\sigma ^{\prime} }_{2})}{\pi {\hslash }^{2}{v}_{F}^{2}}ln\sqrt{\frac{{w}^{2}}{|(\varepsilon -{\sigma ^{\prime} }_{1})(\varepsilon -{\sigma ^{\prime} }_{2})|}+1,}\\ {\sigma ^{\prime} }_{2}={y}_{2}\delta -{y}_{2}(1-{y}_{2}){\delta }^{2}\frac{{{\rm{\Omega }}}_{1}(\varepsilon -{\sigma ^{\prime} }_{1})}{\pi {\hslash }^{2}{v}_{F}^{2}}ln\sqrt{\frac{{w}^{2}}{|(\varepsilon -{\sigma ^{\prime} }_{1})(\varepsilon -{\sigma ^{\prime} }_{2})|}+1}.\end{array}$$

where $${\rm{sign}}(\varepsilon -{\sigma ^{\prime} }_{1})=-\,{\rm{sign}}(\varepsilon -{\sigma ^{\prime} }_{2}),$$ and

26 $$\begin{array}{c}{\tilde{G}}_{01,01}(\varepsilon )=-\,\frac{{{\rm{S}}}_{1}(\varepsilon -{\sigma ^{\prime} }_{2})}{\pi d{\hslash }^{2}{v}_{F}^{2}}ln\sqrt{\frac{{w}^{2}}{(\varepsilon -{\sigma ^{\prime} }_{1})(\varepsilon -{\sigma ^{\prime} }_{2})}-1}-i\frac{{{\rm{S}}}_{1}(\varepsilon -{\sigma ^{\prime} }_{2})}{2d{\hslash }^{2}{v}_{F}^{2}},\\ {\tilde{G}}_{02,02}(\varepsilon )=-\,\frac{{{\rm{S}}}_{1}(\varepsilon -{\sigma ^{\prime} }_{1})}{\pi d{\hslash }^{2}{v}_{F}^{2}}ln\sqrt{\frac{{w}^{2}}{(\varepsilon -{\sigma ^{\prime} }_{1})(\varepsilon -{\sigma ^{\prime} }_{2})}-1}-i\frac{{{\rm{S}}}_{1}(\varepsilon -{\sigma ^{\prime} }_{1})}{2d{\hslash }^{2}{v}_{F}^{2}},\end{array}$$

27 $$\begin{array}{c}\begin{array}{rcl}{\sigma ^{\prime} }_{1} & = & {y}_{1}\delta -{y}_{1}(1-{y}_{1}){\delta }^{2}\frac{{{\rm{\Omega }}}_{1}(\varepsilon -{\sigma ^{\prime} }_{2})}{\pi {\hslash }^{2}{v}_{F}^{2}}ln\sqrt{\frac{{w}^{2}}{|(\varepsilon -{\sigma ^{\prime} }_{1})(\varepsilon -{\sigma ^{\prime} }_{2})|}-1,}\\ {\sigma ^{\prime\prime} }_{1} & = & -{y}_{1}(1-{y}_{1}){\delta }^{2}\frac{{{\rm{\Omega }}}_{1}(\varepsilon -{{\sigma }{^{\prime} }}_{2})}{2{\hslash }^{2}{v}_{F}^{2}},\\ {\sigma ^{\prime} }_{2} & = & {y}_{2}\delta -{y}_{2}(1-{y}_{2}){\delta }^{2}\frac{{{\rm{\Omega }}}_{1}(\varepsilon -{\sigma ^{\prime} }_{1})}{\pi {\hslash }^{2}{v}_{F}^{2}}ln\sqrt{\frac{{w}^{2}}{|(\varepsilon -{\sigma ^{\prime} }_{1})(\varepsilon -{\sigma ^{\prime} }_{2})|}-1,}\end{array}\\ \begin{array}{ccc}{\sigma ^{\prime\prime} }_{2} & = & -{y}_{2}(1-{y}_{2}){\delta }^{2}\frac{{{\rm{\Omega }}}_{1}(\varepsilon -{\sigma ^{\prime} }_{1})}{2{\hslash }^{2}{v}_{F}^{2}},\end{array}\end{array}$$

where $${\rm{sign}}(\varepsilon -{\sigma ^{\prime} }_{1})={\rm{sign}}(\varepsilon -{\sigma ^{\prime} }_{2}).$$

In Eqs (24–27), ${\sigma ^{\prime} }_{i}$ and ${\sigma ^{\prime\prime} }_{i}$ are the real and imaginary parts of the coherent potentials ${\sigma }_{i}$, $i=1,\,2$.

The analysis of formulas (24 and 25) shows that, at the ordering of impurity atoms, the gap $\eta |\delta |$ in width centered at the point $y\delta$ arises in the energy spectrum of graphene. The energies $\varepsilon$ corresponding to the energy gap edges are determined from the equations: $\varepsilon -{\sigma ^{\prime} }_{1}=0$, $\varepsilon -{\sigma ^{\prime} }_{2}=0$. In the considered approximation of weak scattering $|\delta /w|\ll 1$, the second terms in the formulas for ${\sigma ^{\prime} }_{1}$ and ${\sigma ^{\prime} }_{2}$ can be neglected. Relation (14) implies that the maximum value of the parameter of ordering equals ${\eta }_{max}=2y,\,y\le 1/2$. For the complete ordering of impurity atoms, the energy gap width is equal to $2y|\delta |$, i.e., it is proportional to the concentration of an impurity $y$ and to the modulus of the difference of the scattering potentials for components of graphene $\delta$. For $y=1/2$, the gap width takes the maximum value equal to $|\delta |$. For $\delta  > 0$ and $\delta  < 0$, the energy gaps lie, respectively, to the right and to the left from the Dirac point on the energy scale. As is seen from formulas (18) and (24 and 25), the density of electron states in the approximation of coherent potential reads $g(\varepsilon )=0$ for this energy region.

Formulas (18) and (26 and 27) imply that, at the energies outside the region of the gap, the density of electron states reads

28 $$g(\varepsilon )=\frac{{{\rm{\Omega }}}_{1}[\varepsilon -({\sigma ^{\prime} }_{1}+{\sigma ^{\prime} }_{2})/2]}{\pi {\hslash }^{2}{v}_{F}^{2}},$$

where ${\sigma ^{\prime} }_{1}$, ${\sigma ^{\prime} }_{2}$ are given by formula (27).

If the Fermi level is located in the gap, then the number of free charge carriers is equal to zero. The qualitative reasoning implies that the electrical conductance of graphene is also zero in this case. This follows also from formula (19) for the electrical conductance ${\sigma }_{\alpha \alpha }$. If the Fermi level falls in the region of the gap, then, as follows from formulas (19) and (24 and 25), the electrical conductance ${\sigma }_{\alpha \alpha }\to 0$ at the ordering of graphene, i.e., the metal–dielectric transition arises.

Let us study the electrical conductance of graphene in the case where the Fermi level is located outside the gap. Substituting formulas (20)–(22) and (27) in formula (19) and passing from the summation over the wave vectors ${\boldsymbol{k}}$ to the integration, we obtain

29 $${\sigma }_{\alpha \alpha }=\frac{2{e}^{2}\hslash {v}_{F}^{2}}{{\pi }^{2}{a}_{0}^{2}d({y}^{2}-\frac{1}{4}{\eta }^{2}){\delta }^{2}},$$

where $d$ is the thickness of graphene.

It is worth to note that the factor $d$ in the denominator on the right-hand side of formula (29) can be omitted, because it is cancelled in the expression for the electrical resistance of graphene.

Thus, the above-presented results imply that the appearance of a gap in the energy spectrum of graphene is related to the ordering of substitutional impurity atoms.

We have shown that, at the ordering of impurity atoms, a gap $\eta |\delta |$ in width centered at the point $y\delta$ arises in the energy spectrum of graphene. The maximum value of the parameter of ordering is ${\eta }_{max}=2y,\,y\le 1/2$. For the complete ordering of impurity atoms, the energy gap width equals $2y|\delta |$, i.e., it is proportional to the impurity concentration $y$ and to the modulus of the difference of the scattering potentials for components of graphene $\delta$. For $y=1/2$, the gap width takes the maximum value equal to $|\delta |$. For the complete ordering of impurity atoms, the different components of graphene are located on different sublattices. In the approximation of coherent potential, this is described by a step potential, whose value depends on the impurity concentration $y$ and the difference of the scattering potentials for components $\delta$. The symmetry of graphene with an ordered substitutional impurity is lower than the symmetry of pure graphene, which is the cause for the appearance of the energy gap.

If the Fermi level falls in the region of such a gap, then the electrical conductance ${\sigma }_{\alpha \alpha }\to 0$ at the ordering of graphene, i.e., the metal–dielectric transition arises.

If the Fermi level is located outside the gap, then, as is seen from formula (29), the electrical conductance increases with the parameter of order $\eta$ according to the relation

30 $${\sigma }_{\alpha \alpha } \sim {({y}^{2}-\frac{1}{4}{\eta }^{2})}^{-1}.$$

At the concentration $y=1/2,$ as the ordering of impurity atoms $\eta \,\to 1,$ the electrical conductance of graphene ${\sigma }_{\alpha \alpha }\to \infty$, i.e., graphene transits in the state of ideal electrical conductance.
